# Carbonic Anhydrase IV Selective Inhibitors Counteract the Development of Colitis-Associated Visceral Pain in Rats

**DOI:** 10.3390/cells10102540

**Published:** 2021-09-26

**Authors:** Elena Lucarini, Alessio Nocentini, Alessandro Bonardi, Niccolò Chiaramonte, Carmen Parisio, Laura Micheli, Alessandra Toti, Valentina Ferrara, Donatello Carrino, Alessandra Pacini, Maria Novella Romanelli, Claudiu T. Supuran, Carla Ghelardini, Lorenzo Di Cesare Mannelli

**Affiliations:** 1 Department of Neuroscience, Psychology, Drug Research and Child Health (NEUROFARBA), Pharmacology and Toxicology Section, University of Florence, Viale Gaetano Pieraccini 6, 50139 Florence, Italy; elena.lucarini@unifi.it (E.L.); carmen.parisio@unifi.it (C.P.); laura.micheli@unifi.it (L.M.); alessandra.toti@unifi.it (A.T.); valentina.ferrara@unifi.it (V.F.); carla.ghelardini@unifi.it (C.G.); 2 Department of Neuroscience, Psychology, Drug Research and Child Health (NEUROFARBA), Pharmaceutical and Nutraceutical Sciences Section, University of Florence, Via Ugo Schiff 6, 50019 Florence, Italy; alessio.nocentini@unifi.it (A.N.); alessandro.bonardi@unifi.it (A.B.); niccolo.chiaramonte@unifi.it (N.C.); novella.romanelli@unifi.it (M.N.R.); claudiu.supuran@unifi.it (C.T.S.); 3 Department of Experimental and Clinical Medicine, Anatomy and Histology Section, University of Florence, Largo Brambilla 3, 50134 Florence, Italy; donatello.carrino@unifi.it (D.C.); alessandra.pacini@unifi.it (A.P.)

**Keywords:** IBDs, visceral hypersensitivity, carbonic anhydrase IV, colon, inflammation

## Abstract

Persistent pain affecting patients with inflammatory bowel diseases (IBDs) is still very difficult to treat. Carbonic anhydrase (CA) represents an intriguing pharmacological target considering the anti-hyperalgesic efficacy displayed by CA inhibitors in both inflammatory and neuropathic pain models. The aim of this work was to evaluate the effect of inhibiting CA IV, particularly when expressed in the gut, on visceral pain associated with colitis induced by 2,4-di-nitrobenzene sulfonic acid (DNBS) in rats. Visceral sensitivity was assessed by measuring animals’ abdominal responses to colorectal distension. Repeated treatment with the selective CA IV inhibitors AB-118 and NIK-67 effectively counteracted the development of visceral pain induced by DNBS. In addition to pain relief, AB-118 showed a protective effect against colon damage. By contrast, the anti-hyperalgesic activity of NIK-67 was independent of colon healing, suggesting a direct protective effect of NIK-67 on visceral sensitivity. The enzymatic activity and the expression of CA IV resulted significantly increased after DNBS injection. NIK-67 normalised CA IV activity in DNBS animals, while AB-118 was partially effective. None of these compounds influenced CA IV expression through the colon. Although further investigations are needed to study the underlying mechanisms, CA IV inhibitors are promising candidates in the search for therapies to relieve visceral pain in IBDs.

## 1. Introduction

Persistent abdominal pain is still an intractable problem in patients affected by inflammatory bowel diseases (IBDs). Although IBD-related symptoms have been classically attributed to intestinal inflammation, 20–50% of patients develop a chronic syndrome characterised by pain, bloating and dysmotility, which also persists after the remission of colitis [1,2,3]. The complex nature of this type of pain makes patients partially responsive to anti-inflammatory drugs, anticonvulsants or antidepressants, which are classically used in the management of pain syndromes [4,5].

Recent advances in the identification and characterisation of novel drug targets have evidenced carbonic anhydrase inhibition as a new approach for designing novel pain-relieving agents [6]. CA inhibitors have been demonstrated to be effective in the treatment of both inflammatory [7,8,9] and neuropathic pain conditions [10,11,12,13,14,15]. CAs are enzymes involved in various physiological reactions including respiration, pH regulation, Na^+^ retention, tumorigenesis, electrolyte secretion and metabolism. Hence, CA inhibitors have long been used as systemic anticonvulsants, anti-glaucoma agents, and for treating altitude sickness [16,17,18,19]. Interestingly, the CA inhibitor acetazolamide has been reported to reduce referred pain caused by abdominal laparoscopic surgery under carbon dioxide insufflation, suggesting that CAs also have a role in visceral pain perception [20]. CA was also found to be strongly increased in the muscles of women with chronic musculoskeletal pain, demonstrating that this class of enzymes is also involved in peripheral signaling modulation [21].

Significant and persistent alterations in both the periphery and central nervous system have been documented in animals subjected to intestinal inflammatory damage [4,22]. Although it is not known whether a dysregulation of CA activity occurs in these processes, the commonalities existing between chronic visceral pain and neuropathic pain [4,23] make CA an interesting target to investigate in the search for new therapies for abdominal pain relief in patients affected by IBD.

Moreover, evidence collected in the last few years from preclinical studies suggest the possibility of developing specific therapies for pain management based on the inhibition of selective Cas’ isoforms involved in particular pain conditions (e.g., selective modulation of CA II/VII activity showed highly improved efficacy compared to acetazolamide in neuropathic pain) [6]. The aim of the present work was to investigate the role of CA IV, which is well-represented throughout the intestine, in the resolution of the intestinal damage and in the development of persistent visceral pain subsequent to colitis induction in rats. For this purpose, the effect of the treatment with newly synthetised CA inhibitors displaying a good affinity to the isoform CA IV, was studied in a rat model of IBD induced by 2,4-dinitrobenzenesulfonic acid (DNBS).

## 2. Materials and Methods

### 2.1. Chemistry

The synthesis of NIK-67 was previously reported by us. Anhydrous solvents and all reagents for the synthesis of AB-118 were purchased from Sigma Aldrich (Milan, Italy). Nuclear magnetic resonance (^1^H-NMR, ^13^C-NMR) spectra were recorded using a Bruker Advance III 400 MHz spectrometer in DMSO-d_6_. Analytical thin-layer chromatography (TLC) was carried out on Sigma Aldrich silica gel F-254 plates. The melting point (mp) was measured in open capillary tubes with a Gallenkamp MPD350.BM3.5 apparatus and is uncorrected. 

### 2.2. Synthesis of 4-(2-(6-Amino-7H-purin-7-yl)ethoxy)benzenesulfonamide AB-118

1,3-Dibromoethane (1.2 eq) was added dropwise to a suspension of *N*,*N*-dimethyl-*N*′-(9*H*-purin-6-yl)formimidamide (0.4 g, 1.0 eq) [6] and K_2_CO_3_ (1.2 eq) in dry dimethylformamide (4 mL) under a nitrogen atmosphere and the reaction mixture was stirred for 1 h at rt. The reaction was quenched with slush and the formed precipitate, that is, 7-(2-bromoethyl)-7*H*-purin-6-amine was filtered-off under vacuo and, without further purification, added to a suspension of *N*′-((4-hydroxyphenyl)sulfonyl)-*N*,*N*-dimethylformimidamide (1.0 eq) [6] and K_2_CO_3_ (1.2 eq) in dry dimethylformamide (4 mL) under a nitrogen atmosphere. The reaction mixture was stirred at 60 °C overnight and thus quenched with slush, and the formed precipitate was filtered-off under vacuo. The obtained raw solid was suspended in HCl 1.25 M in methanol (10 mL) in a sealed tube and the reaction mixture was stirred at 80 °C until the consumption of the starting material (TLC monitoring). The solution was concentrated under vacuo and the residue triturated with acetone. The obtained hydrochloric salt was treated with a NaHCO_3_ aqueous solution to give the pure title compound AB-118 with a 56% yield; mp 280–281 °C; silica gel TLC R_f_ 0.08 (MeOH/CH_2_Cl_2_ 10% *v*/*v*); δ_H_ (400 MHz, DMSO-*d_6_*): 4.35 (t, *J* = 4.8, 2H, CH_2_), 4.88 (t, *J* = 4.8, 2H, CH_2_), 7.04 (s, 2H, exchange with D_2_O, NH_2_), 7.05 (d, *J* = 8.8, 2H, Ar-H), 7.23 (s, 2H, exchange with D_2_O, SO_2_NH_2_), 7.74 (d, *J* = 8.8, 2H, Ar-H), 8.22 (s, 1H, Ar-H), 8.33 (s, 1H, Ar-H); δ_C_ (100 MHz, DMSO-*d_6_*): 46.5, 68.6, 111.9, 115.4, 128.6, 137.7, 147.4, 152.4, 153.1, 160.8, 161.1; *m*/*z* (ESI negative) 333.0 [M − H].

### 2.3. Animals

For all the experiments described below, Male Sprague-Dawley rats (Envigo, Varese, Italy) weighing approximately 220–250 g at the beginning of the experimental procedure, were used. Animals were housed in CeSAL (Centro Stabulazione Animali da Laboratorio, University of Florence) and used at least 1 week after their arrival. Four rats were housed per cage (size 26 × 41 cm); animals were fed a standard laboratory diet and tap water ad libitum, and kept at 23 ± 1 °C with a 12 h light/dark cycle, with lights on at 7 a.m. All animal manipulations were carried out according to the Directive 2010/63/EU of the European parliament and of the European Union Council (22 September 2010) on the protection of animals used for scientific purposes. The ethical policy of the University of Florence complies with the Guide for the Care and Use of Laboratory Animals of the US National Institutes of Health (NIH Publication No. 85–23, revised 1996; University of Florence assurance number: A5278-01). Formal approval to conduct the described experiments was obtained from the Animal Subjects Review Board of the University of Florence. Experiments involving animals have been reported according to ARRIVE guidelines [24]. All efforts were made to minimise animal suffering and to reduce the number of animals used.

### 2.4. Induction of Colitis and Drug Administration

Colitis was induced in accordance with the method previously described [25] with minor changes. Briefly, during a short period of anaesthesia with isoflurane (2%), 30 mg of 2,4-dinitrobenzenesulfonic acid (DNBS) in 0.25 mL of 50% ethanol was administered intrarectally via a polyethylene PE-60 catheter inserted 8 cm proximal to the anus. Control rats received 0.25 mL of saline solution. Both AB-118 and NIK-67 (75 mg kg^−1^) were suspended in CMC 1% and the respective suspensions were administered once daily in DNBS-treated animals (10 mL kg^−1^ per os) starting from the day of DNBS injection and the treatment continued for 14 days. Tests were performed on day 8 and 15, 24 h after the last treatment. The dose of AB-118 and NIK-67 was chosen on the basis of previous studies showing CA inhibitors’ anti-hyperalgesic effects [8,12,13]. The substances were administered once daily for 14 days to evaluate the effect of CA IV inhibition on visceral pain development (Day 8, acute inflammatory phase of colitis) and on visceral pain persistence (Day 15, post-inflammatory phase of colitis) [4].

### 2.5. Assessment of Visceral Sensitivity by Viscero-Motor Response (VMR)

The viscero-motor response (VMR) to colorectal balloon distension were used as an objective measure of visceral sensitivity in animals. Two EMG electrodes were sutured into the external oblique abdominal muscle under deep anaesthesia and exteriorised dorsally (Christianson and Gebhart, 2007). VMR assessment were carried out under light anaesthesia (Isoflurane 2%). A lubricated latex balloon (length: 4.5 cm), assembled to an embolectomy catheter and connected to a syringe filled with water was used to perform colo-rectal distension. The balloon was inserted into the colon and positioned 6.5 cm from the anus and was filled with increasing volumes of water (0.5, 1, 2, 3 mL). The electrodes were relayed to a data acquisition system and the corresponding EMG signals subsequent to colo-rectal stimulation were recorded, amplified and filtered (Animal Bio Amp, AD Instruments, Colorado Springs, CO, USA), digitised (PowerLab 4/35, AD Instruments, Colorado Springs, CO, USA), analysed and quantified using LabChart 8 (ADInstruments, USA). To quantify the magnitude of the VMR at each distension volume, the area under the curve (AUC) immediately before the distension (30 s) was subtracted from the AUC during the balloon distension (30 s) and responses were expressed as the percentage increase from the baseline. The time that elapsed between two consecutive distension was 5 min.

### 2.6. Assessment of Visceral Sensitivity by Abdominal Withdrawal Reflex (AWR)

The behavioural responses to colorectal distension (CRD) were assessed in the animals by measuring the abdominal withdrawal reflex (AWR), a semi-quantitative score described previously in conscious animals [26]. Briefly, rats were anaesthetised with isoflurane, and a lubricated latex balloon (length: 4.5 cm) attached to polyethylene tubing, assembled to an embolectomy catheter and connected to a syringe filled with water was inserted through the anus into the rectum and descending colon of adult rats. The tubing was taped to the tail to hold the balloon in place. Then rats were allowed to recover from the anaesthesia for 30 min. AWR measurement consisted of visual observation of animal responses to graded CRD (0.5, 1, 2, 3 mL) by blinded observers who assigned AWR scores: no behavioural response to colorectal distention (0); immobile during colorectal distention and occasional head clinching at stimulus onset (1); mild contraction of the abdominal muscles but absence of abdomen lifting from the platform (2); observed strong contraction of the abdominal muscles and lifting of the abdomen off the platform (3); arching of the body and lifting of the pelvic structures and scrotum (4).

### 2.7. Histological Analysis of Colonic Damage

The animals were sacrificed 15 days after DNBS injection, and the colon was macroscopically analysed to assess the damage. To quantify the injury, a macroscopic damage score (MDS) was assigned to each colon in accordance with the criteria previously reported [25] The macroscopic criteria were the following: presence of adhesions between the colon and other intra-abdominal organs (0–2); consistency of colonic faecal material (indirect marker of diarrhoea; 0–2); thickening of colonic wall (mm); presence and extension of hyperaemia and macroscopic mucosal damage (0–5). A colon segment (around 1 cm) was fixed in 4% paraformaldehyde for 24 h, dehydrated in alcohol, encased in paraffin, and finally cut into 5 μm sections. Microscopic evaluations were carried out on haematoxylin/eosin-stained sections of full-thickness samples obtained from the distal colon. Digitalised images were collected at 10, 20, and 40× magnification by a Leica DMRB light microscope equipped with a DFC480 digital camera (Leica Microsystems, Milan, Italy), and analysed quantitatively using the ImageJ software.

### 2.8. Kinetic Determination of CA IV Activity in Colon Samples and AB-118 Inhibition Profile

Colon samples collected from the animals were stored at −80 °C until analysis. Tissue samples were lysed by immersion in liquid nitrogen and disrupted with a mechanical process using marble mortar. The resulting lysed samples were diluted with the addition of five time their volume of buffer (20 mM Hepes, pH 7.5) and then sonicated in order to solubilise the enzymes. After centrifugation at 14,000 rpm for 3 min the supernatant containing only soluble enzyme was removed and the precipitate was resuspended in the buffer with SDS 0.2% to extract membrane proteins. After centrifugation at 14,000 rpm for 3 min the protein concentration in the supernatant was determined spectrophotometrically at 280 nm. The treated and diluted extract enzymatic activity related to CAs was measured by evaluating the CA catalysed CO_2_ hydration reaction by an Applied Photophysics Stopped-Flow instrument. Phenol red (at a concentration of 0.2 mM) was used as an indicator, working at the absorbance maximum of 557 nm, with 20 mM Hepes, pH 7.5 as a buffer, and 20 mM Na_2_SO_4_ (for maintaining constant ionic strength), and by following the initial rates of the CA-catalysed CO_2_ hydration reaction for a period of 10–100 s. The CO_2_ concentration ranged from 1.7 to 17 mM for the determination of the kinetic parameters and inhibition constants. The uncatalysed rates were determined in the same manner and subtracted from the total observed rates. Stock solution of AB-118 (0.1 mM) was prepared in distilled-deionised water and dilutions up to 0.01 nM were done thereafter with the assay buffer. Inhibitor and enzyme solutions were preincubated together for 15 min at room temperature prior to assay to allow for the formation of the E-I complex. The inhibition constants were obtained by non-linear least-squares methods using PRISM 3 and the Cheng–Prusoff equation [27] and represent the mean of at least three different determinations. All CA isoforms were recombinant enzymes obtained in-house [28]. Enzyme concentrations in the assays were in the range of 3–16 nM.

### 2.9. Immunohistochemical Analysis of CA IV Expression 

For immunoreactions, tissue was cut into 5 µm slices and dried on glass slides prior to deparaffinisation with xylol and rehydration in a descending alcohol series (100, 95, 75, and 50%). The slices were incubated overnight at 4 °C with a rabbit anti-human/mouse CA IV antisera validated in rats [29], diluted 1:500 in PBS/5% BSA (Sigma-Aldrich, Milan Italy). The following day, slides were washed thrice with PBS, and then incubated in blocking solution for 1 h with goat anti-rabbit secondary antibodies labelled with Alexa Fluor 568. To stain nuclei, sections were incubated with DAPI in PBST for 5 min, at room temperature in the dark. After three washes in PBS and a final wash in distilled water, slices were mounted using Fluoromount-G™ Mounting Medium (Thermo Fisher Scientific, Milan, Italy) as mounting medium. Digitalised images were collected at 100×, 200×, or 400× total magnification by a motorised Leica DM6000B microscope equipped with a DFC350FX. Quantitative analysis of CA IV-related immunofluorescence intensity was performed by collecting independent fields (8–12 for each animal) from the mucosa and the myenteric plexi, by using ImageJ (NIH, Bethesda, MD, USA). The value relative to the background was subtracted from the value obtained from the analysed area and the results were expressed as a percentage of the control group.

### 2.10. Statistics

All measurements were made by researchers blinded to the animal treatments. Data were analysed using “Origin 9” software (OriginLab, Northampton, MA, USA) by one or two-way analysis of variance (ANOVA) with a Bonferroni post-test, with *p* < 0.05 or 0.01 considered statistically significant, respectively. Results are shown as means ± standard error of the mean (SEM) of n assessments depending on the experiment.

## 3. Results

### 3.1. The CA IV Inhibitors AB-118 and NIK-67 Counteracted the Development of Visceral Pain Induced by Colitis in Rats

AB-118 and NIK-67 [30] were assayed as inhibitors of a panel of human CAs, which are isoforms of the ubiquitous, cytosolic and off-target CAs I and II, the membrane-bound and target CA IV and the transmembrane tumour-associated CA IX, using a stopped-flow CO_2_ hydrase assay (Table 1).

Both inhibitors showed a selective action against the target CA IV over the other tested isozymes. In detail, AB-118 and NIK-67 inhibited CA IV with inhibition constants (K_I_s) of 28.7 and 2.3 nM, respectively. Most relevantly, AB-118 and NIK-67 inhibited the target CA six and twenty-six times more efficiently than the most physiologically important, and thus off-target isoform CA II.

**Figure 1:**
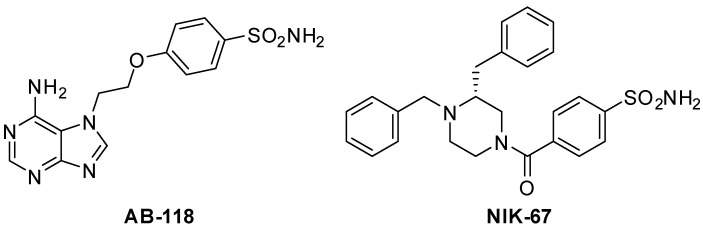
Structures of AB-118 and NIK-67.

**Figure 2:**
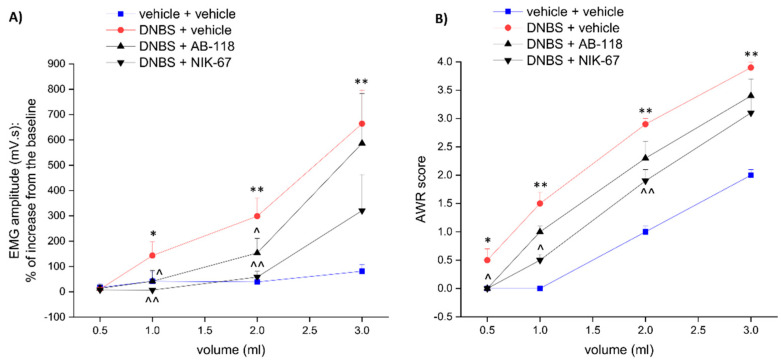
Effect of AB-118 and NIK-67 on the development of visceral pain in the acute phase of colitis induced by DNBS in rats. Tests were performed 8 days after DNBS injection, 24 h after the last administration of the CAIV inhibitor, AB−118 or NIK−67 (75 mg kg^−1^ p.o.). Visceral sensitivity was assessed by measuring (**A**) the animals visceral motor response (VMR) and (**B**) the abdominal withdrawal response (AWR) to colon rectal distension (0.5, 1, 2, 3 mL balloon inflation). Each value is the mean ± S.E.M. and represents the mean of 5 rats per group. * *p* < 0.05 and ** *p* < 0.01 vs. vehicle + vehicle treated animals. ^ *p* < 0.05 and ^^ *p* < 0.01 vs. DNBS + vehicle-treated animals.

**Figure 3:**
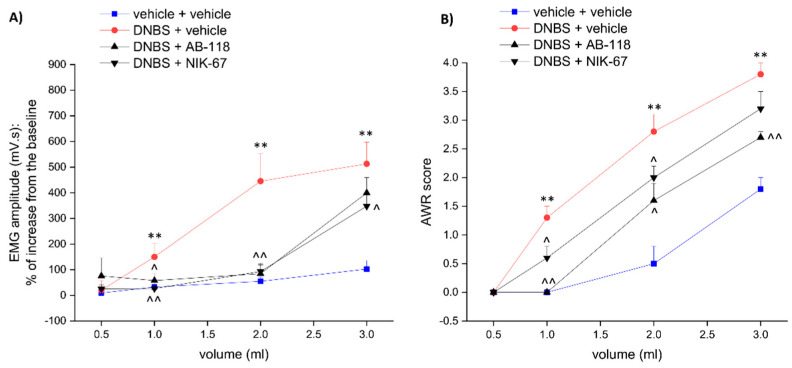
Effect of AB-118 and NIK-67 on the persistence of visceral pain in the post-inflammatory phase of colitis induced by DNBS in rats. Tests were performed 15 days after DNBS injection (30 mg in 0.25 mL EtOH 50%), 24 h after the last administration of the CAIV inhibitor, AB-118 or NIK-67 (75 mg kg^−1^ p.o.). Visceral sensitivity was assessed by measuring (**A**) the animals visceral motor response (VMR) and (**B**) the abdominal withdrawal response (AWR) to colon rectal distension (0.5, 1, 2, 3 mL balloon inflation). Each value is the mean ± S.E.M. and represents the mean of 5 rats per group. ** *p* < 0.01 vs. vehicle + vehicle treated animals. ^ *p* < 0.05 and ^^ *p* < 0.01 vs. DNBS + vehicle-treated animals.

The CA IV inhibitors AB-118 and NIK-67 (Figure 1) were orally administered (75 mg kg^−1^) in animals for 14 days, starting from the day of DNBS injection. Visceral pain was evaluated in the acute inflammatory phase (day 8) and in the post-inflammatory phase (day 15) of colitis (Figure 2 and Figure 3, respectively), 24 h after the last administration of the compounds, by measuring the visceral motor response (VMR; A) and the abdominal withdrawal response (AWR; B) to colorectal distension. DNBS injection caused a persistent increase in visceral sensitivity in the animals, which showed a significant enhancement in both the VMR and AWR response to colorectal distension either on day 8 (Figure 2A,B, respectively) or day 15 (Figure 3A,B, respectively).

The treatment with AB-118 or NIK-67 counteracted the increase in visceral sensitivity caused by DNBS injection. Both on day 8 and 15, animals receiving DNBS + AB-118 showed a VMR significantly lower than those treated with DNBS + vehicle when 1- and 2-mL stimuli were applied, while no relief was detected for the higher volume of distension (3 mL; Figure 2A and Figure 3A, respectively). As a result of the treatment with AB-118, he AWR to both 2- and 3-mL volumes of distension was also significantly reduced at each time tested (Figure 2B and Figure 3B).

NIK-67 displayed a slightly higher efficacy than AB2-118 in reducing the VMR in response to colorectal distension on day 8 after DNBS injection (Figure 2A). On day 15, the VMR evoked by either 1,2 or 3 mL-stimulus was significantly lower in DNBS animals administered with NIK-67 in respect to those injected with the vehicle (Figure 3A). Nevertheless, NIK-67 was less effective than AB-118 in reducing the AWR in DNBS-treated animals (Figure 2B and Figure 3B).

### 3.2. Effect of the Repeated Administration of AB2-118 and NIK-67 on Colon Damage

**Figure 4:**
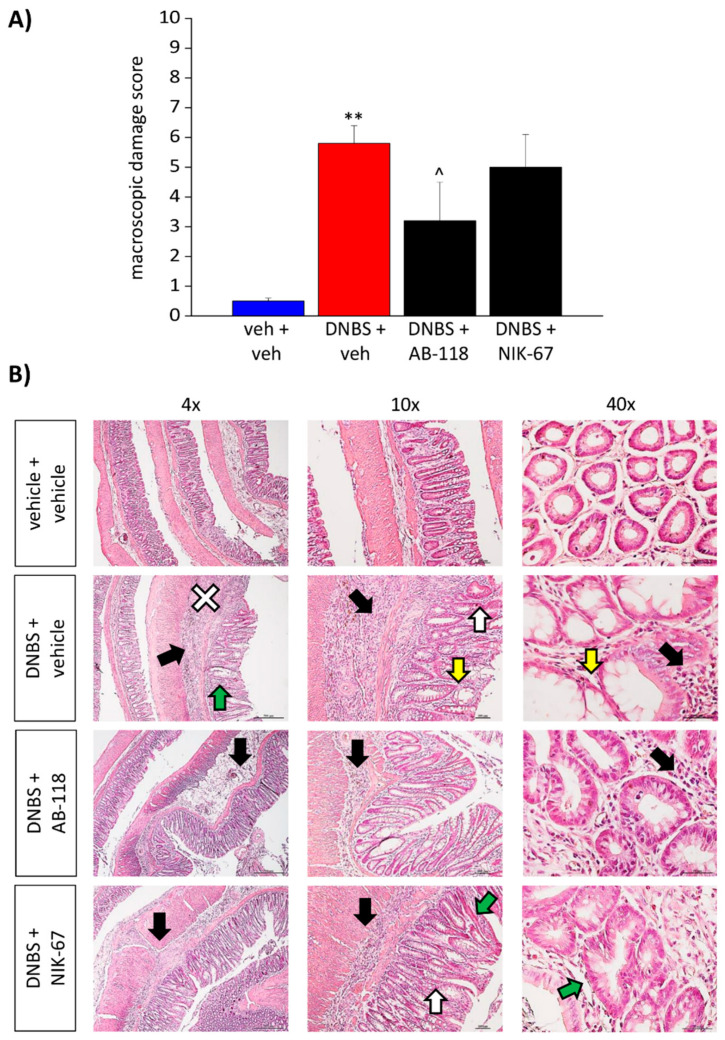
Effect of AB-118 and NIK-67 on the colon damage induced by the injection of DNBS. Colon damage was assessed 15 days after the intrarectal injection of DNBS. A macroscopic damage score (MDS) was assigned to each animal based on the presence of adhesions between colon and other intra-abdominal organs (0–2); the consistency of colonic faecal material (indirect marker of diarrhoea; 0–2); the presence and extension of hyperaemia and macroscopic mucosal damage (0–5); the thickening of colonic wall (mm) (**A**). Microscopic evaluations were performed on sections stained with haematoxylin and eosin (scale bar: 4×, 10× and 40×) with particular attention to: inflammatory cell infiltration (black arrow), loss of epithelium surface (white cross), goblet cell hyperplasia and mucus hypersecretion (yellow arrow), irregular crypts (green arrow), epithelial hyperplasia (visible as crypt elongation, with goblet cell loss; white arrow) (**B**). Each value is the mean ± S.E.M. and represents the mean of 5 rats per group. ** *p* < 0.01 vs. vehicle + vehicle-treated animals. ^ *p* < 0.05 vs. DNBS + vehicle-treated animals.

The animals were sacrificed 15 days after DNBS injection, and the colon was analysed both macroscopically (Figure 4A) and microscopically (Figure 4B) to assess the damage. The macroscopic damage score (MDS) showed by DNBS-treated animals appeared significantly higher than that of controls. The repeated treatment with AB-118 was able to significantly reduce the macroscopic damage induced by DNBS, while NIK-67 did not show a protective effect (Figure 4A).

From a microscopic point of view, the colon of DNBS-treated animal appeared significantly thickened with inflammatory infiltration. Despite the restoration of the tunica mucosa, the colon of DNBS-treated animals showed spot loss of the epithelial surface, probably resulting from healing processes on previous deep ulcers. The crypts were elongated with irregular diameters and shapes, and goblet cells showed hyperplasia and mucus hypersecretion. In the group of animals receiving DNBS + AB-118, the tunica mucosa was mostly restored, the crypts showed a structure and shape like those of the controls, though they still appeared elongated and enlarged. In this group, the presence of inflammatory infiltrate was significantly reduced and almost exclusively limited to the submucosa, although the colonic wall still thickened. By contrast, no significative difference was observed between the animals treated with DNBS + NIK-67 and those receiving DNBS + vehicle (Figure 4B). In DNBS animals treated with either AB-118 or NIK-67, no goblet cell hyperplasia or mucus hypersecretion was observed.

### 3.3. Effect of the Repeated Administration of AB2-118 and NIK-67 on Intestinal CA IV Activity

The CO_2_ hydratase activity of the treated extracts, associated only to CA IV, was measured by a stopped-flow kinetic assay on day 15. In fact, CA IV features two disulphide linkages that confer resistance to denaturation by exposure to detergents such as SDS (0.2–5%) used to extract membrane proteins. In contrast, this detergent concentration was reported to inactivate most other CA isoforms [32]. Thus, SDS-resistance enabled determination of the CA IV contribution to total hydratase activity in tissues containing multiple CA isoforms.

**Figure 5:**
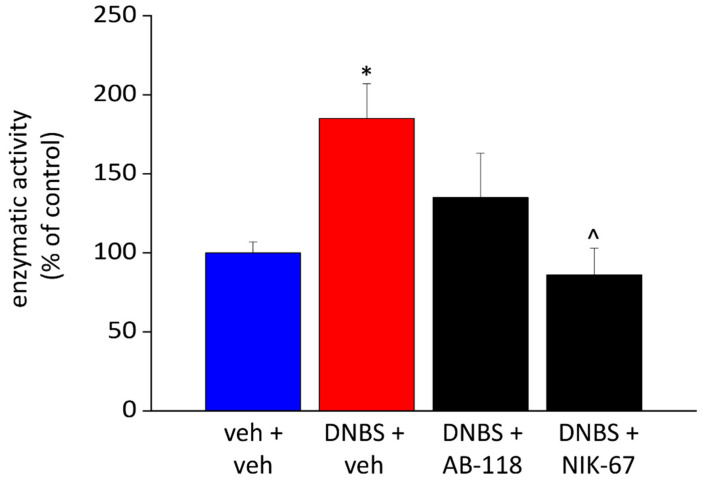
Effect of DNBS injection and AB-118 or NIK-67 repeated administration on colon CA IV activity. The CA IV enzymatic activity was assessed 15 days after DNBS injection by measuring the rate of the CO_2_ hydration reaction catalysed by the treated extracts using an Applied Photophysics Stopped-Flow instrument. The enzymatic activity was expressed as percentage of controls. Each value is the mean ± S.E.M. and represents the mean of 5 rats per group. * *p* < 0.05 vs. vehicle + vehicle treated animals. ^ *p* < 0.05 vs. DNBS + vehicle treated animals.

The enzymatic activity of CA IV, expressed as percentage of the controls, resulted significantly increased in DNBS treated animals (Figure 5). The repeated administration of NIK-67 after DNBS injection induced a significant decrease in CA IV activity, which was comparable to that of the controls. Even the enzymatic activity of CA IV in the colon of animals treated with AB-118 showed a trend to reduction, but without reaching the statistical significance (Figure 5).

### 3.4. The Expression of CA IV Resulted Increased through the Colon Wall of DNBS-Treated Rats

**Figure 6:**
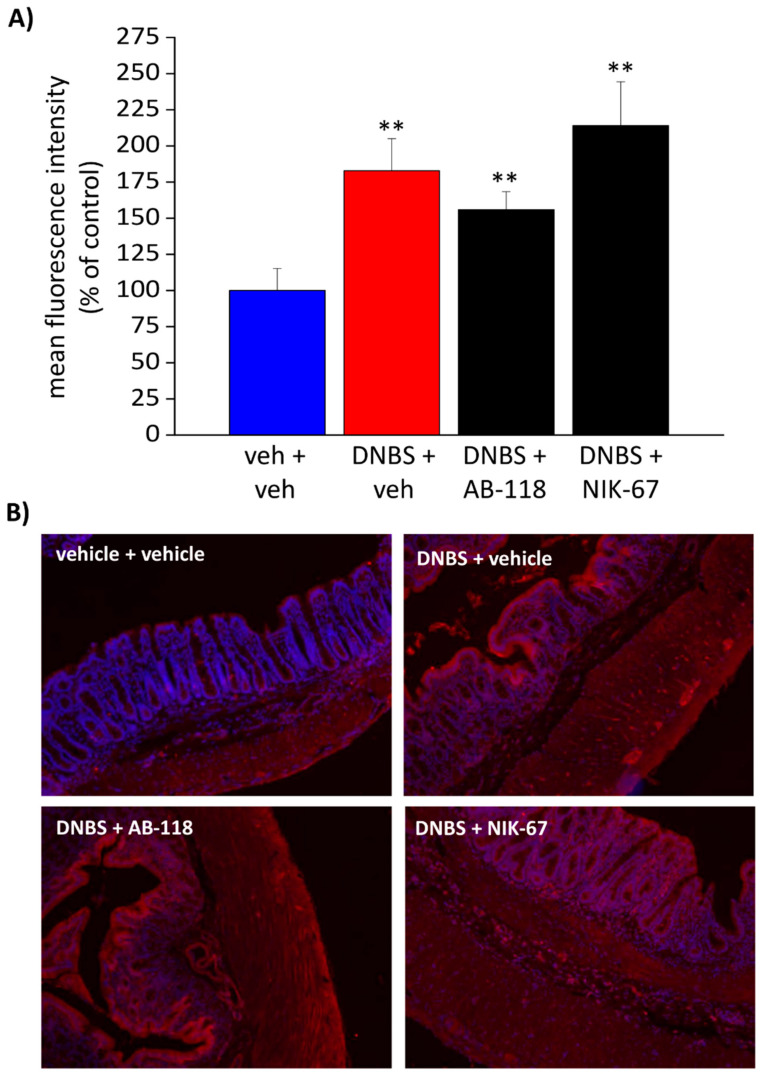
Effect of DNBS injection and AB-118 or NIK-67 administration on CA IV expression in colon mucosa. The distribution of CA IV on colon sections was evaluated by immunofluorescence on tissue sections. The mean fluorescence intensity relative to the entity of CA IV expression in the mucosa was expressed as a percentage of the control (**A**). Representative images of the colon mucosa were reported (scale bar: 10×; **B**). The values are the means ± SEM of the measurements of individual animals (at least five images each) from the different experimental groups. ** *p* < 0.01 vs. vehicle + vehicle group.

**Figure 7:**
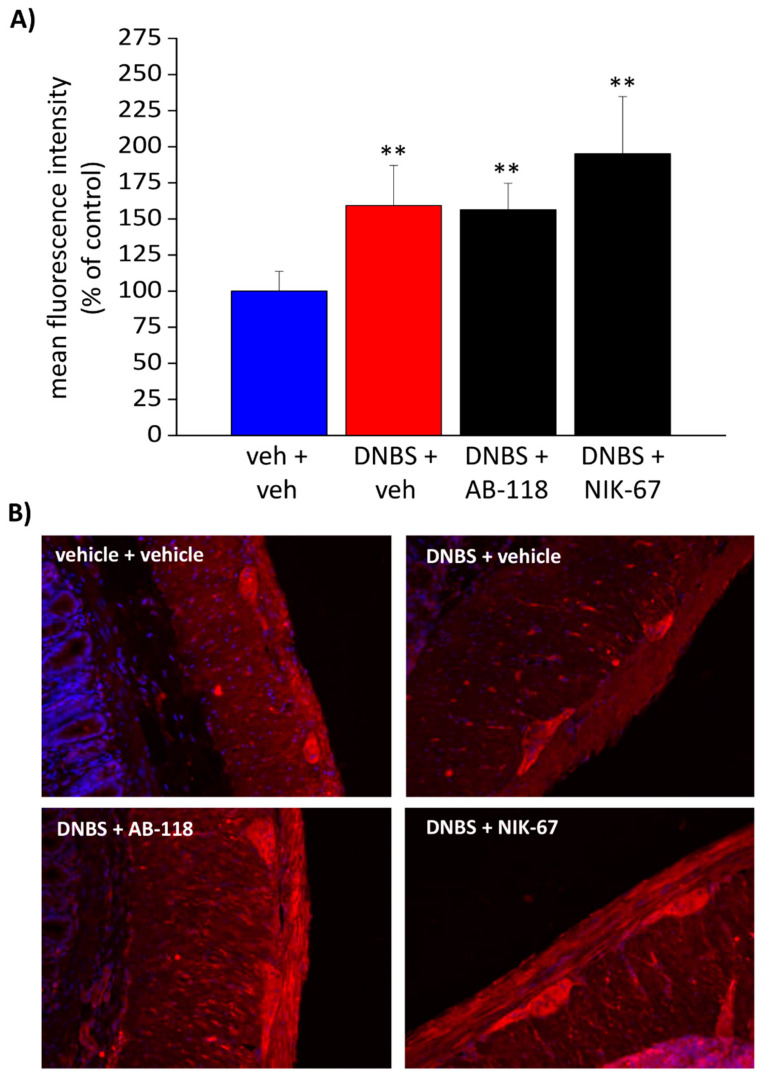
Effect of DNBS injection and AB-118 or NIK-67 administration on CA IV expression in colon myenteric plexus. The distribution of CA IV on colon sections was evaluated by immunofluorescence on tissue sections. The mean fluorescence intensity relative to the entity of CA IV expression in the myenteric plexus was expressed as a percentage of the control (**A**). Representative images of the colon myenteric plexus were reported (scale bar: 40×; **B**). The values are the means ± SEM of the measurements of individual animals (at least five images each) from the different experimental groups. ** *p* < 0.01 vs. vehicle + vehicle group.

Immunofluorescence with a specific antibody was performed to study CA IV expression through the colonic wall (Figure 6 and Figure 7). A predominant CA IV immunoreactivity was detected in the mucosa and the myenteric plexi (Figure 6B and Figure 7B). As a result of colitis, the mean fluorescence intensity relative to CA IV expression resulted significantly augmented in both the mucosal epithelial layer (Figure 6A) and the myenteric plexus (Figure 7A) of all the animals treated with DNBS. The administration of either the CA IV inhibitors, AB-118 or NIK-67, in DNBS animals did not alter the expression of the enzyme (Figure 6A and Figure 7A). Higher magnification imagines of the colon mucosa (scale bar: 20×) from different animals were reported in the (Supplementary Materials Figure S1), showing an intense stain of the apical plasma membranes of non-goblet epithelial cells. An increase in CA IV expression occurs in DNBS-treated animals, while the administration of CA IV inhibitors does not influence the expression of the enzyme through the mucosa (Figure S1).

## 4. Discussion

The present work highlighted the involvement of CA IV in the development of visceral pain induced by colitis. CA inhibitors AB-118 and NIK-67 were selected for this study because of their potent (K_I_s of 28.7 nM and 2.3 nM, respectively) and selective (greater than 6- and 26-fold, respectively) inhibitory action against the target CA IV, over the main off-target isozymes (CAs I and II). Both the CA IV inhibitors, AB-118 and NIK-67, effectively counteracted the increase in visceral sensitivity subsequent to DNBS injection in rats. Moreover, AB-118 showed a protective effect on colon damage. Indeed, either the enzymatic activity or the expression of CA IV (in colon epithelial and myenteric plexi) significantly increased after DNBS injection. NIK-67, which was 10-fold more effective than AB-118 (K_I_ of 2.3 nM vs. 28.7 nM), normalised CA IV activity in DNBS animals, while AB-118 was partially effective. Anyway, both compounds did not influence CA IV expression through the colon.

Previous studies demonstrated abundant CA IV expression in the distal small intestine and large intestine, where CA IV was localised to the apical membrane of the epithelium. CA IV was additionally found in submucosal capillary endothelium of all gastrointestinal regions [33]. Likewise, we observed that CA IV was highly expressed in the epithelial layer. In addition, we detected a great amount of CA IV through the myenteric ganglia of the enteric nervous system, which plays a pivotal role in inflammatory and nociceptive processes [34,35]. This evidence offers a direct link between the CA and visceral sensitivity regulation in the gut, though the role played by CA in the enteric nervous system needs to be further investigated.

Interestingly, the amount of CA IV, as well as its enzymatic activity, was found to be increased in the colon of DNBS-treated animals 14 days after damage induction, suggesting the over-functionality of CA IV as a pathological characteristic of colitis and supporting the therapeutic use of CA inhibitors. Although the reduced activity of CA, mainly CA I, was observed in the acute phase of inflammatory diseases like colitis, the role of carbonic anhydrases, and particularly that of CA IV, in the post-inflammatory alterations induced by colitis have not been investigated before. The pain-relieving profile of AB-118 and NIK-67 paralleled a reduction in CA IV activity. Enzyme expression was not affected by inhibitors, although this response should not be expected with the repeated use of enzyme inhibitors that commonly favour an up-regulation.

AB-118 treatment favoured tissue healing from the inflammatory damage, along with pain relief, though it only partially reduced the activity of CA IV. The protective effect on intestinal damage of AB-118 with respect to NIK-67 might be related to the more effective inhibitory action of CA IX (K_I_ of 87.2 nM vs. 418.5 nM), which has been associated to acidosis and damage in inflamed tissues [7]. On the other hand, NIK-67, which presents a very low K_I_ for CA IV, counteracted pain in a manner that was independent of tissue healing, suggesting a direct effect of NIK-67 on visceral sensitivity regulation. This data strengthens the hypothesis of the active involvement of CA IV in the development and persistence of pain induced by colitis. Besides, this evidence indicates that other CAs might be more involved in the regulation of tissue healing than CA IV.

Goblet cells are mucin-secreting intestinal cells that form the mucus layer that protects the mucosal surface. Of note, an increase in H^+^ has been observed in different inflammatory diseases, such as pulmonary hypertension and rheumatoid arthritis [36,37] where acetazolamide showed protective anti-inflammatory effects by a dual mechanism of macrophage carbonic anhydrase inhibition and systemic metabolic acidosis [38]. Recent findings highlighted inflammation-induced uncoupling of CA and sodium–hydrogen antiporter 1 (NHE-1) in experimental colitis, which might lead to an intracellular accumulation of H^+,^ resulting in acidosis and necrosis in the inflamed colon [37]. The colon surface, flattened during active colitis, presents colonic crypt elongation and, eventually, goblet cells hyperplasia during the recovery phase of acute colitis. In this phase, the inhibition of carbonic anhydrase activity has been demonstrated to enhance the recovery from colitis in mice by directly stimulating colonic epithelial cells proliferation [39]. Local acidosis might provide a causal link between inflammatory diseases, CAs deregulation and the persistence of pain. In a recent work, Potenzieri et al. reported that oxaliplatin-induced neuropathy occurs through impairment of haemoglobin proton buffering and is reversed by carbonic anhydrase inhibitors [40].

A decrease in mucus secretion through the gastrointestinal tract has been reported to be associated with the inhibition of carbonic anhydrase activity [41]. Accordingly, the inhibition of CA IV mediated by AB-118 and NIK-67 counteracted mucus hyperserection observed in DNBS-treated animals during the post-inflammatory phase of colitis. As an important part of the innate immune system, the mucus barrier helps to maintain a mutualistic immune relationship between the host and bacteria and to reduce the activation of subepithelial lymphocytes [42]. The imbalanced mucus barrier may play an important role in the disease progression of IBD: an affected mucus barrier is evident in patients with ulcerative colitis (UC), and at the same time, the hyperproduction of mucins and abnormal glycosylation are typical in Crohn’s disease (CD). This phenomenon has been linked to diffferent types of mucins’ expression in patients with UC and CD [43]. Therefore, the effect of CA IV on mucus secretion is an aspect that need to be further investigated in the context of specific intestinal diseases as well as in physiological conditions.

Another interesting point is that CAs are also expressed by the pathogenic as well as non-pathogenic bacteria present in the intestinal microflora, where the role of CAs is beginning to be understood. Apart from pH regulation and adaptation to various niches in which bacteria live, CAs probably participate in biosynthetic processes in which bicarbonate or CO_2_ are substrates [44]. Nevertheless, the inhibition and activation of bacterial CAs have not been exploited from either a pharmacological or an environmental viewpoint. CA IV inhibitors, once orally administered, might directly influence microbiota metabolism. On the other hand, since CA IV are particularly expressed in the apical plasma membrane of epithelial cells [45], CAs inhibition may alter the microenvironment where microbiota resides, indirectly affecting its composition and/or metabolism. This evidence assumes even more relevance if we consider that microbiota has a key role in the regulation of visceral sensitivity [41], both in physiological and pathological conditions, as attested by the literature [46,47].

As already mentioned, carbonic anhydrase inhibition showed beneficial effects in painful conditions with different aetiologies, such as neuropathy and osteoarthritis, attesting to the relevance of CA manipulation in the regulation of pain sensitivity [7,8,9,12]. Regarding the involvement of CAs in visceral pain, the only evidence comes from studies investigating the beneficial effects of CAs inhibitors in the treatment of post-operative pain induced by laparoscopy [32]. In the central nervous system, CA activation transforms GABA-mediated inhibition (Cl conductance) into excitation due to increased HCO_3_^−^ flux through the GABAA receptor channel [48,49]. Such synaptic transformation allows GABA-releasing interneurons to act as either excitation filters or amplifiers of the neuronal network. In this context, CA inhibitors can reduce the bicarbonate-dependent depolarisation of GABAA receptors, showing analgesic effects [6]. Peripheral GABA receptors regulate colonic afferent excitability and visceral nociception [50]. It is possible that a derangement in GABA receptor function, mediated by increased levels of HCO_3_^−^ in the gut, might be involved in post-inflammatory visceral pain persistence, where an increase in the activity of CAs was found.

Yet, spinal carbonic anhydrase has been reported to contribute to nociceptive hyperreflexia induced by pentobarbital and midazolam and to a lesser extent with propofol [51]. Intrathecal acetazolamide has been demonstrated to possess anti-allodynic effects in the presence of a chloride dysregulation [52], as occurs in neuropathic conditions, where the hypofunction of the potassium-chloride co-transporter KCC2 has been reported [53]. The KCC2 downregulation-mediated impairment of spinal cord Cl^−^ homeostasis has been associated with chronic stress-induced visceral hypersensitivity and neonatal cystitis-induced visceral pain in rats [54,55]. Therefore, the modulation of visceral pain mediated by CA inhibitors might involve both the periphery and the spinal cord, which represents an important regulatory station along the pathways of visceral pain signalling [4].

The current therapeutic approaches to inflammatory bowel disease (IBD) symptoms, including psycho-nutritional and pharmacological treatments (bulking-agents, antidiarrheals, antispasmodics, antidepressants) are almost ineffective against abdominal pain, which highly impacts the patient’s quality of life) [1]. The mechanisms involved in visceral pain persistence are heterogeneous and all the actors involved in visceral sensitivity modulation serve other important physiological functions. The identification of a target involved in orchestrating the events underlying pain and selectively modulable to obtain pain relief without causing undesirable side effects might represent a turning point in the therapy of visceral pain. Although further mechanistic investigations are needed to elucidate the role of CA in either gut physiology or visceral sensitivity regulation, altogether, the evidence collected to date make CA IV a promising target in the search for therapies to relieve persistent abdominal pain in IBD patients.

## Figures and Tables

**Table 1:** Inhibition data of human CA isoforms I, II, IV and IX with NIK-67 and AB-118 by a stopped-flow CO_2_ hydrase assay. Mean from three different assays by a stopped-flow technique (errors were in the range of ±5–10% of the reported values).

Compound	K_I_ (nM) [31]^.^			
	CA I	CA II	CA IV	CA IX
NIK-67 [30]	380.2	60.7	2.3	418.5
AB-118	783.7	174.3	28.7	87.2

## Data Availability

The data presented in this study are available on request from the corresponding author. The data are not publicly available due to privacy restrictions.

## Supplementary Materials

The following are available online at https://www.mdpi.com/article/10.3390/cells10102540/s1, Figure S1: Effect of DNBS injection and AB-118 or NIK-67 administration on CA IV expression in colon mucosa.
