# Supplementary material for: Carbonic Anhydrase IV Selective Inhibitors Counteract the Development of Colitis-Associated Visceral Pain in Rats
Source: Cells. 2021 Sep 26;10(10):2540. doi: 10.3390/cells10102540 (PMC8533707; doi:10.3390/cells10102540)
Supplement: Supplementary file 1 [file cells-10-02540-s001.zip › cells-1330588-supplementary.pdf]

Supplementary materials

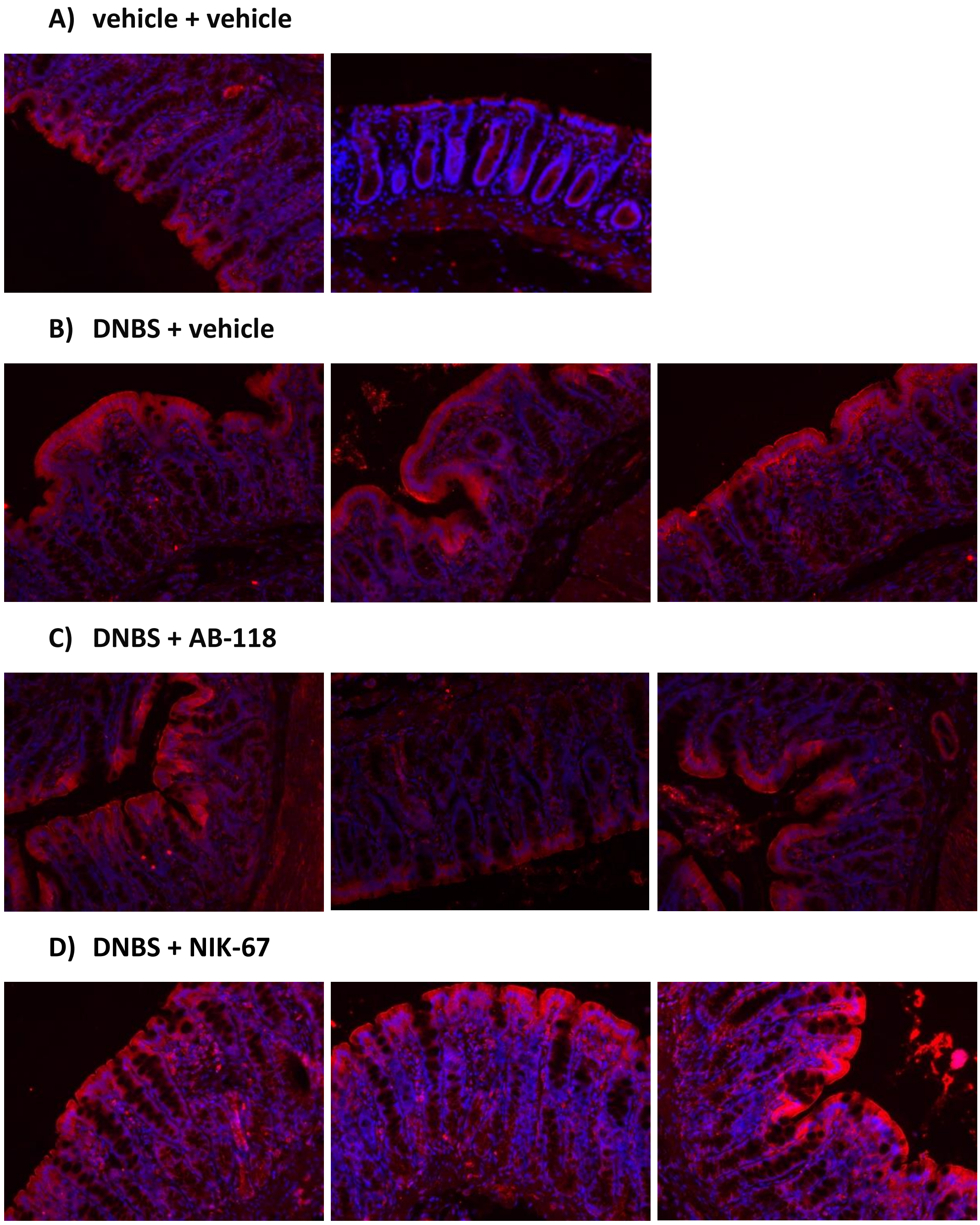

Figure S1. Effect of DNBS injection and AB-118 or NIK-67 administration on CA IV expression in colon mucosa. The distribution of CA IV on colon sections was evaluated by immunofluorescence on tissue sections. Representative images of the colon mucosa were reported (scale bar: 20×) for different animals of vehicle + vehicle group (**A**), DNBS + vehicle group (**B**), DNBS + AB-118 group (**C**) and DNBS + NIK-67 group (**D**), respectively.
